# Connexin Restrains EMT by Dual-Domain Mechanisms to Preserve Epithelial Identity

**DOI:** 10.21203/rs.3.rs-7501684/v1

**Published:** 2025-11-27

**Authors:** Bo Ma, Zhaodan Ding, Guangyan Wang, Sumin Gu, Jean X. Jiang

**Affiliations:** 1 Department of Biochemistry and Structural Biology, University of Texas Health Science Center, San Antonio, TX 78229-3900; 2 The First Affiliated Hospital of Xi’an Jiaotong University, Xi’an, China

## Abstract

Epithelial–mesenchymal transition (EMT) contributes to fibrotic disease in multiple organs and underlies post-surgical complications such as posterior capsule opacification (PCO), the leading cause of vision loss following cataract surgery. The molecular mechanisms that preserve epithelial integrity and restrain EMT remain poorly defined. Connexins are conventionally regarded as gap junction proteins mediating intercellular communication, but here we identify connexin 50 (Cx50) as a previously unrecognized key regulator of EMT. Using in vitro, ex vivo, and in vivo chick and mouse models, we show that transforming growth factor-β2 (TGF-β2) downregulates Cx50 and induces EMT markers including α-smooth muscle actin and fibronectin, changes reversed by Cx50 overexpression. Mechanistically, the extracellular E2 domain of Cx50 mediates adhesive interactions that limit epithelial migration and EMT, while the C-terminal domain stabilizes β-catenin–E-cadherin complexes and prevents TGF-β2–driven E-cadherin loss and β-catenin nuclear translocation. Dominant-negative E2 mutants abolish protection, and Cx50 knockout mice exhibit accelerated EMT after extracapsular lens extraction. These findings redefine connexins beyond their classical role as gap junction proteins, establishing Cx50 as a dual-domain regulator of EMT and epithelial plasticity with implications for fibrotic responses in ocular and other epithelial tissues.

## Introduction

Cataract is the leading cause of blindness worldwide, and surgery is the only effective treatment. However, posterior capsular opacification (PCO), also known as secondary cataract, is the most common complication following cataract surgery [1] and can significantly reduce postoperative vision. The underlying mechanism of PCO is not yet fully understood, but it is believed to result from residual lens epithelial cells (LECs) that, under the stimulation of cytokines, undergo epithelial-mesenchymal transition (EMT), synthesize extracellular matrix (ECM), and ultimately contribute to PCO formation [2]. Among these processes, EMT of lens epithelial cells is a key step in PCO development [1b, 3]. During the transdifferentiation of epithelial cells into the myofibroblast phenotype, they lose tight and adherens junction complexes, including ZO-1 and E-cadherin. Additionally, β-catenin dissociates from the cell membrane and translocates to the nucleus [4]. This process leads to a gradual transition from an epithelial phenotype, characterized by predominant E-cadherin expression, to a mesenchymal phenotype marked by increased Ncadherin expression. As β-catenin enters the nucleus, it acts as a co-transcription factor, promoting the transcription of genes involved in multiple EMT-related signaling pathways. Ultimately, this leads to the reorganization of the epithelial cell cytoskeleton and the expression of α-SMA protein and other extracellular matrix proteins, marking the occurrence of EMT [5].

Connexins (Cxs) form both gap junctions and hemichannels, facilitating communication between cells, and between cells and the extracellular environment, respectively. These channels play crucial roles in cell growth, development, and differentiation, and their dysfunction contributes to multiple pathological conditions [6]. Three Cx subtypes are expressed in the lens: Cx43 and Cx50 in lens epithelial cells and Cx50 and Cx46 in lens fibers. Studies have shown that functional gap junctions in lens fiber cells play an important role in maintaining lens homeostasis and transparency [7]. However, the role of lens Cxs in EMT and PCO development remains largely elusive. The non-channel functions of Cxs, particularly Cx50, have recently garnered increasing attention [8]. We previously demonstrated the channel-independent role of Cx50 in promoting lens cell differentiation into lens fiber cells [9]. Our recent study also showed that Cx50 interacts with integrins, which opens hemichannels and facilitates nutrient and antioxidant transport into lens fiber cells [10]. Moreover, we identified a unique role of Cx50 in lens cell-cell adhesion, which is essential for lens epithelial-to-fiber differentiation and is independent of its channel-forming ability [11]. Additionally, we found that Cx50, rather than Cx43 or Cx46, promotes cell adhesion through its second extracellular loop (E2) domain [12].

Previous research has shown that increasing cell-cell adhesion can inhibit cell migration [13]. Maintaining E-cadherin expression at the cell membrane and preventing the nuclear translocation of β-catenin are also effective in suppressing epithelial cell migration [14]. Migration and proliferation of LEC are early events in PCO development [15], and targeting LEC migration has been proposed as a potential strategy to suppress PCO progression [16]. Given that Cx50 promotes cell-cell adhesion, we hypothesize that Cx50 may inhibit LEC EMT and PCO development by strengthening cell-cell adhesion.

In this study, we employed a combination of *in vitro, ex vivo*, and *in vivo* models to investigate the role and molecular mechanisms of Cx50 in PCO development. These included a TGF-β–induced EMT model using primary chick lens epithelial cells (CLCs), an *ex vivo* wound-repair model using embryonic chick lens capsules, and both *ex vivo* capsule explants and *in vivo* extracapsular lens extraction (ECLE) mouse models. To dissect Cx50 function, we utilized dominant-negative adhesion mutants targeting the Cx50 E2 domain as well as Cx50 knockout (KO) mice. Across all models, we consistently observed an inhibitory role of Cx50 in EMT and PCO progression and demonstrated that the Cx50 E2 domain suppresses EMT by promoting cell–cell adhesion. Furthermore, we showed that Cx50 retains β-catenin in the cytosol through direct interaction via its C-terminal (CT) domain, thereby preventing its nuclear translocation, a hallmark event in EMT.

## Results

### Cx50 expression decreases, while EMT markers increase in PCO models

**Figure 1.**
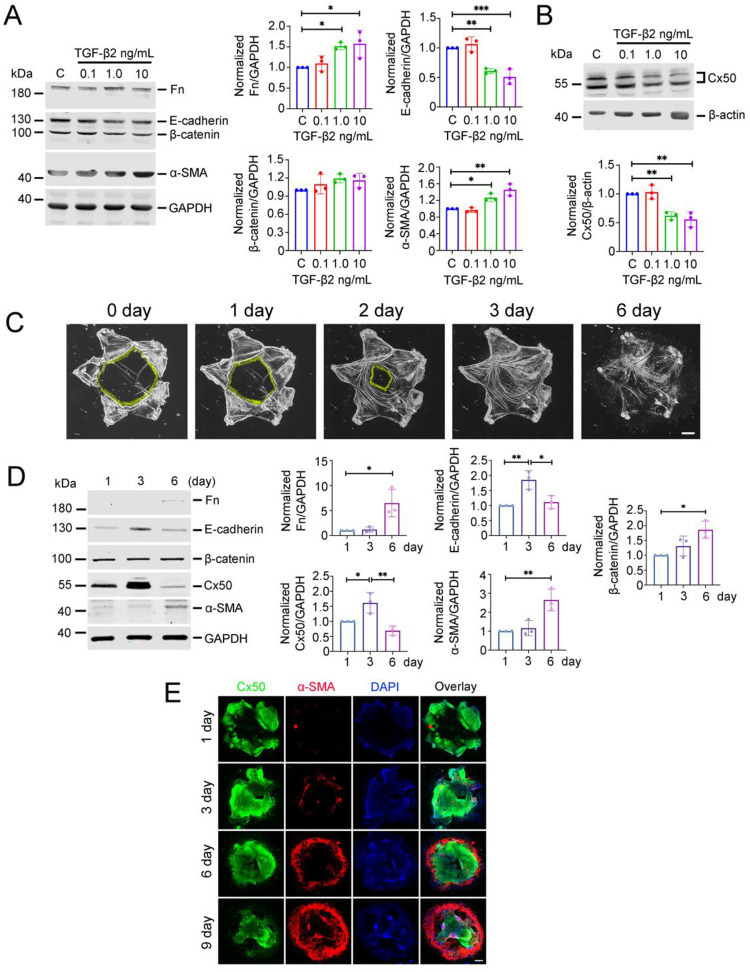
Cx50 decreases in both *in vitro* and *ex vivo* embryonic chick lens EMT models. **(A, B)** Primary chick lens cell cultures were treated with different concentrations of TGF-β2, and cell lysates were immunoblotted for fibronectin (Fn), E-cadherin, β-catenin, α-SMA, or GAPDH (**A,** left panels). Crude membrane preparations were immunoblotted for Cx50 or β-actin (**B,** upper panels). Band intensities were quantified using NIH ImageJ (**A,** right graphs; **B,** lower graph). Data are presented as the mean ± SD. (n = 3). *, P < 0.05; **, P < 0.01; ***, P < 0.001. One-way ANOVA with Tukey’s multiple comparison test was used for statistical analysis. **(C, D)** The chick embryonic lens ex vivo wound-repair model was used, and lens epithelial cell migration at different time points was imaged **(C)**. Fn, E-cadherin, β-catenin, α-SMA, Cx50 and GAPDH were detected by immunoblotting (**D,** left panels) and band intensities were quantified (right graphs). Scale bar = 500 μm. The data are presented as the mean ± SD (n = 3). *, P < 0.05; **, P < 0.01 (one-way ANOVA with Tukey’s multiple comparison test). **(E)** Extracts of chick embryonic lens capsules were co-immunostained with Cx50 (green) and anti-α-SMA (red) antibodies, with DAPI counterstaining (blue). Scale bar = 500 μm.

We first used an *in vitro* PCO model by treating primary CLCs with increasing concentrations of TGF-β2 (0, 0.1, 1.0, 10 ng/mL), as previously reported [17]. Western blot analysis showed a dosedependent increase in fibronectin (Fn) and α-smooth muscle actin (α-SMA), accompanied by a decrease in E-cadherin and Cx50 expression. (Figure 1A, B). Notably β-catenin expression remained largely unchanged under these conditions. To further validate these findings, we employed an *ex vivo* embryonic chick lens wound-repair model of PCO [18]. In this model, lens epithelial cells migrated to the center of the posterior capsule from day 0 to day 2. By day 3, the posterior capsule was almost fully covered with cells, by day 6, it appeared wrinkled and contracted (Figure 1C). Western blotting showed significantly increased expression of fibronectin (Fn), α-SMA, and β-catenin on day 6 (Figure 1D). Interestingly, Cx50 and E-cadherin expression transiently increased on day 3 but significantly decreased by day 6. Co-immunostaining further confirmed that Cx50 expression progressively declined from day 1 to day 9, while α-SMA expression increased during the same period (Figure 1E). Initially, α-SMA localization was restricted to proliferating peripheral cells, but later became more prominent in the central region of the capsule. Collectively, these results suggest that Cx50 expression is inversely correlated with the progression of EMT in LEC during PCO development.

### Cx50 E2 domain-mediated adhesion inhibits lens epithelial cell (LEC) migration and EMT in PCO models

**Figure 2.**
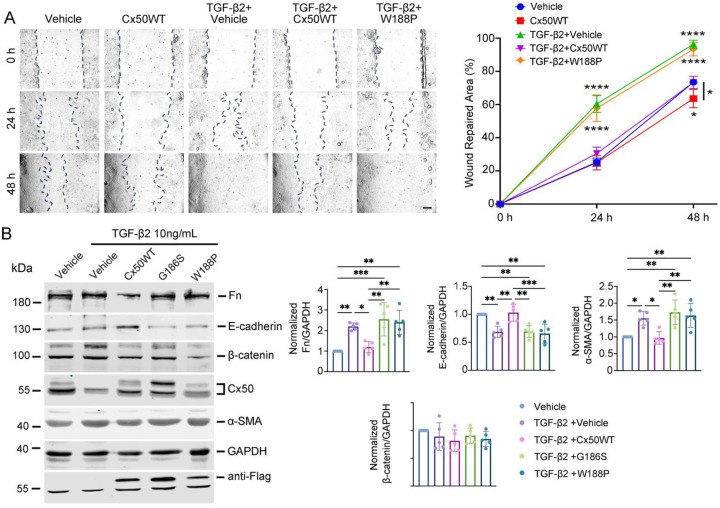
Cx50 inhibits LEC migration and EMT marker expression via the Cx50 E2 domain. **(A)** Primary chicken lens cells were infected with recombinant retroviruses expressing WT Cx50 or the Cx50 mutant W188P and treated with 10 ng/mL TGF-β2. A wound healing assay was conducted, and images were captured at 0, 24, and 48 hours (left panels). LECs migration was quantified by measuring the wound-repaired area using NIH ImageJ (right graph). Scale bar = 50 μm. Data are presented as the mean ± SD (n = 3). *, P < 0.05; **, P < 0.01; ***, P < 0.001, ****, P < 0.0001 (Two-way ANOVA). **(B)** Primary chicken lens cell cultures were infected with recombinant retroviruses expressing WT Cx50 or the Cx50 mutants G186S or W188P and treated with 10 ng/mL TGF-β2. Fn, E-cadherin, β-catenin, Cx50, α-SMA, and GAPDH were detected by immunoblotting with their corresponding antibodies. Exogenous Cx50 was detected by an antiFlag antibody (left panels). Band intensities were quantified using NIH ImageJ (right graphs). Data are presented as mean ± SD (n = 3). *, P < 0.05; **, P < 0.01; ***, P < 0.001 (One-way ANOVA with Tukey’s multiple comparison test).

Cell-cell adhesion plays a critical role in suppressing EMT development [19], and Cx50 has been shown to mediate lens cell adhesion through its extracellular E2 domain [12]. To investigate this function in the context of PCO, we performed a wound-repair assay using primary CLCs expressing exogenous WT Cx50 or adhesion-deficient, dominant-negative Cx43 E2 dominant mutants (G186S and W188P) via recombinant RCAS(A) retroviral infection. Immunofluorescence with an anti-FLAG antibody confirmed comparable expression levels of exogenous WT Cx50 and Cx50 E2 mutants in CLCs (**Figure S1**). As expected, CLCs treated with 10 ng/mL TGF-β2 migrated significantly faster than untreated control (Figure 2A). However, CLCs expressing exogenous WT Cx50 migrated significantly more slowly than the vehicle-infected controls after 48 hours. This inhibitory effect on migration was attenuated by the W188P mutant, indicating the importance of the E2 domain in Cx50-mediated adhesion. Notably, even in the absence of TGF-β2, CLCs expressing exogenous WT Cx50 exhibited significantly reduced migration compared to vehicle controls, suggesting that Cx50 overexpression alone can suppress CLC migration. Western blot analysis revealed that TGF-β2 treatment significantly increased fibronectin (Fn) and α-SMA expression and decreased E-cadherin levels (Figure 2B), consistent with EMT induction. Overexpression of WT Cx50 reversed these effects, reducing Fn and α-SMA while increasing E-cadherin levels. In contrast, CLC expressing adhesion-deficient Cx50 E2 mutants (G186S and W188P) failed to suppress EMT marker expression. Expression of β-catenin did not show any significant changes. Together, these results show that Cx50 inhibits LEC migration and EMT progression, and this effect is mediated by its E2-domin-dependent cell-cell adhesion function.

**Figure 3.**
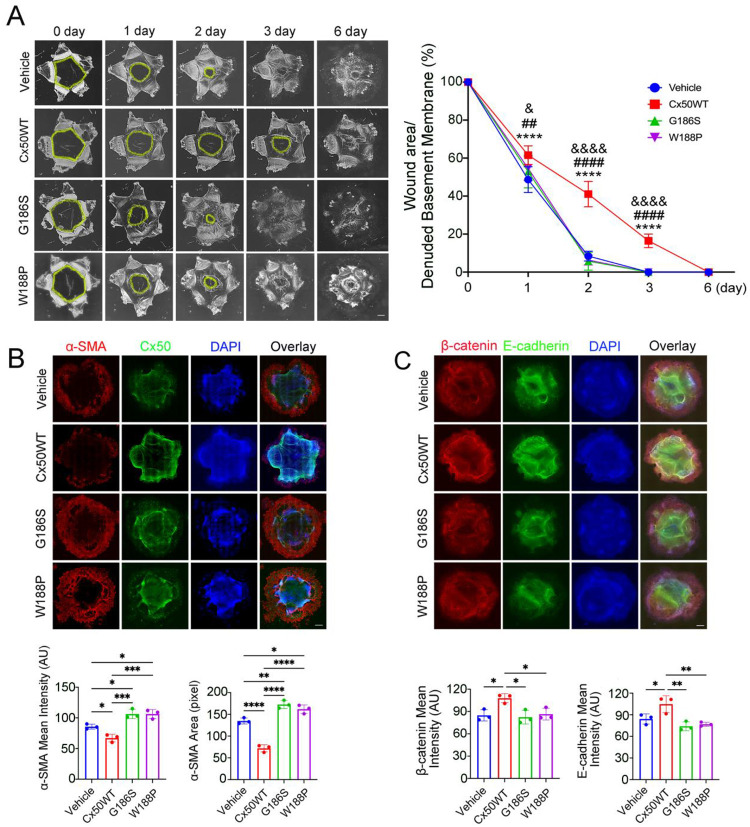
Cx50 inhibits LEC migration and EMT marker expression via the Cx50 E2 domain in an *ex vivo* embryonic chick lens wound repair model. **(A)** Isolated chick embryonic lens capsular bags were infected with recombinant retroviruses expressing WT Cx50 or Cx50 mutants G186S and W188P. Lens epithelial cell migration was imaged at different time points (left panels). The wound area and denuded basement membrane were quantified using NIH ImageJ (right graph). Statistical comparisons: *, Vehicle vs. WT Cx50; ^#^, WT Cx50 vs. G186S; ^&^, WT Cx50 vs. W188P. Data are presented as mean ± SD (n = 3). ****, P < 0.0001; ^##^, P < 0.01, ^####^, P < 0.0001; ^&^, P < 0.05, ^&&&&^, P < 0.0001 (Two-way ANOVA). **(B)** Chick embryonic lens capsule extracts were immunostained for Cx50 (green) and α-SMA (red), with DAPI counterstaining (blue) (upper panels). Mean fluorescence intensities were quantified using NIH ImageJ (lower graphs). **(C)** Capsules extracts of chick embryonic lens infected with recombinant retroviruses expressing WT Cx50 or Cx50 mutants were immunostained for E-cadherin (green) and β-catenin (red), counterstained with DAPI (blue) (upper panels). Mean fluorescence intensities were quantified using NIH ImageJ (lower graphs). For **B, C,** data are presented as mean ± SD (n = 3). *, P < 0.05; **, P < 0.01; ***, P < 0.001; ****, P < 0.0001. (One-way ANOVA with Tukey’s multiple comparison test). Scale bar = 500 μm.

To further investigate the inhibitory roles of Cx50 in LEC migration and EMT, we used an *ex vivo* embryonic chick lens wound-repair model. LECs were infected with recombinant RCAS(A) retroviruses carrying WT Cx50 or Cx50 E2 domain mutants (G186S and W188P). Co-immunostaining confirmed the expression of WT Cx50 and Cx50 E2 mutants in nearly all cells, as detected by anti-FLAG antibody (**Figure S2**). During the wound-repair process, LECs migrated from the anterior to the posterior capsule between day 0 and day 2, and by day 3, they had completely filled the capsular cavity (Figure 3A). LECs expressing exogenous WT Cx50 migrated significantly more slowly than vehicle-injected control, as indicated by the persistent cavity remaining at the center of the capsule on day 3. In contrast, LECs expressing the Cx50 E2 mutants G186S or W188P, migrated significantly faster than those expressing WT Cx50. Notably, LECs expressing these mutants migrated faster than the vehicle controls, likely due to dominant-negative disruption of cell adhesion mediated by endogenous Cx50. Similarly, immunostaining showed decreased α-SMA expression in LECs expressing WT Cx50, whereas LECs expressing G186S or W188P, exhibited significantly higher α-SMA levels compared to both vehicle and WT Cx50-expressing cells (Figure 3B). Additionally, β-catenin and E-cadherin levels were significantly elevated in WT Cx50-expressing LECs relative to vehicle controls (Figure 3C). This effect was reversed in cells expressing Cx50 E2 mutants, which showed markedly reduced β-catenin and E-cadherin levels. Together, these results further demonstrate that Cx50 suppress LEC migration and EMT through its E2 domain-mediated cell adhesion function in an *ex vivo* embryonic lens woundrepair model.

### Deletion of Cx50 in the mouse lens enhances TGF-β2-induced EMT and promotes β-catenin nuclear localization

**Figure 4.**
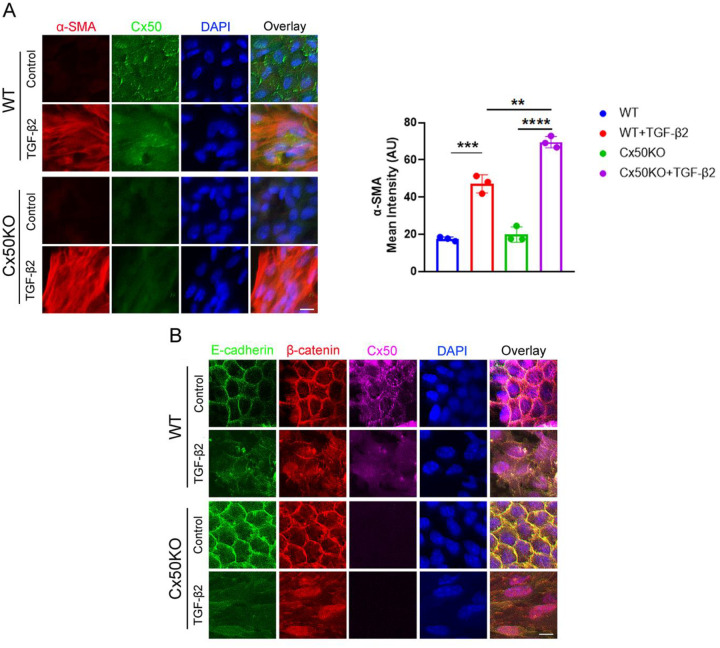
Deletion of Cx50 enhances TGF-β2-induced EMT and promotes β-catenin nuclear localization. *Ex vivo* lens capsule explants from WT and Cx50 KO mice were cultured and treated with 10 ng/mL TGF-β2. **(A)** Mouse lens capsules were immunostained for Cx50 (green) and α-SMA (red), with DAPI nuclear counterstaining (blue). Mean fluorescence intensities were quantified using NIH ImageJ (right graphs). Data are presented as mean ± SD (n = 3). **, P < 0.01; ***, P < 0.001, ****, P < 0.0001 (One-way ANOVA with Tukey’s multiple comparison test). Scale bar = 10 μm. **(B)** Mouse lens capsule explants were immunostained for E-cadherin (green), β-catenin (red), and Cx50 (purple), with DAPI nuclear counterstaining (blue). Scale bar = 10 μm.

We used an *ex vivo* PCO model with capsule explants isolated from mouse lenses, as previously described [20], and employed Cx50 KO mouse model to assess the role of Cx50 in EMT. Double and triple co-immunostaining revealed that α-SMA expression increased while Cx50 expression decreased in lens explants of WT mouse following treatment with 10 ng/mL TGF-β2 (Figure 4A). Notably, α-SMA expression was significantly higher in Cx50 KO mice compared to WT control. Co-immunostaining showed that Cx50 co-localized with both E-cadherin (**Figure S3A**) and β-catenin (**Figure S3B**), and that E-cadherin co-localized with β-catenin (**Figure S3C**). Triple immunostaining further confirmed that β-catenin, E-cadherin, and Cx50 were co-localized at cell membrane (Figure 4B). Following TGF-β2 treatment, expression of E-cadherin and Cx50 was reduced, and β-catenin translocated from the membrane to the nucleus. However, some β-catenin and E-cadherin remained on cell membrane. In contrast, in Cx50 KO mice, nearly all β-catenin translocated to the nuclear, and no E-cadherin was detected on cell membrane. The results indicate that the deletion of Cx50 enhances TGF-β2-induced EMT and promotes β-catenin nuclear localization.

### Cx50 E2-mediated cell adhesion increases E-cadherin expression and reduces β-catenin nuclear localization

**Figure 5.**
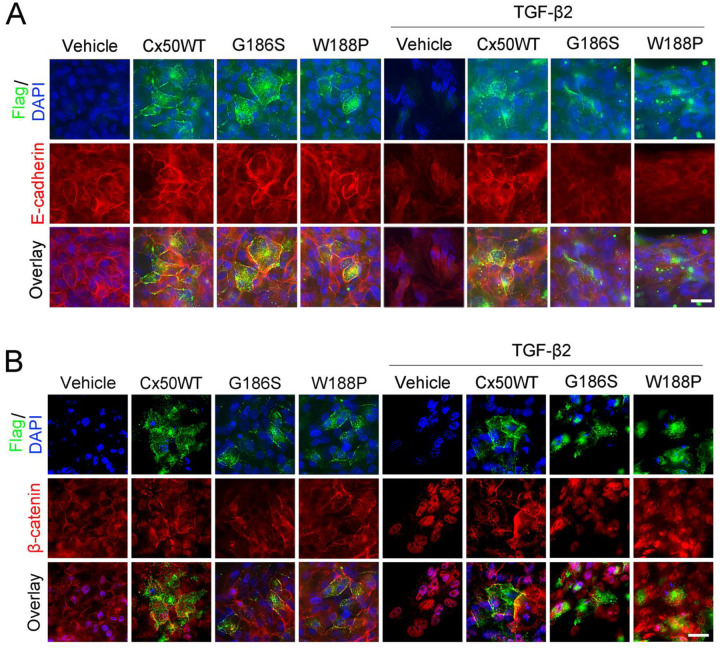
Cx50 inhibits E-cadherin loss and β-catenin nuclear localization mediated by the Cx50 E2 domain. Primary chicken lens cells were infected with recombinant retroviruses expressing WT Cx50 or Cx50 E2 mutants (G186S or W188P) and treated with 10ng/mL TGF-β2. **(A)** Co-immunostaining was performed for exogenous Cx50 (Flag, green) and E-cadherin (red), with DAPI nuclear counterstaining (blue). Scale bar = 20 μm. **(B)** Co-immunostaining was performed for exogenous Cx50 (Flag, green) and β-catenin (red), with DAPI counterstaining (blue). Scale bar = 20 μm.

Primary CLCs were infected with recombinant RCAS(A) expressing either WT Cx50 or Cx50 E2 domain mutants (G186S and W188P). Co-immunostaining showed that exogenous Cx50 detected via its FLAG tag, co-localized with E-cadherin and β-catenin on the cell membrane. Similarly, endogenous Cx50 was also found to colocalize with E-cadherin and β-catenin in CLCs (**Figure S4A, B**). Treatment with 10 ng/mL TGF-β2 reduced E-cadherin levels and promoted β-catenin translocation of to the nucleus. In contrast, overexpression of WT Cx50 preserved E-cadherin expression and maintained β-catenin localization at the membrane. However, the Cx50 E2 mutants (G186S and W188P) reduced E-cadherin expression and promoted β-catenin nuclear localization (Figure 5A, B). These results indicate that Cx50 promotes E-cadherin expression and inhibits β-catenin nuclear localization through E2 domain-mediated cell adhesion.

### Cx50 prevents β-catenin nuclear translocation through its direct interaction with the Cx50 cytosolic CT domain.

**Figure 6.**
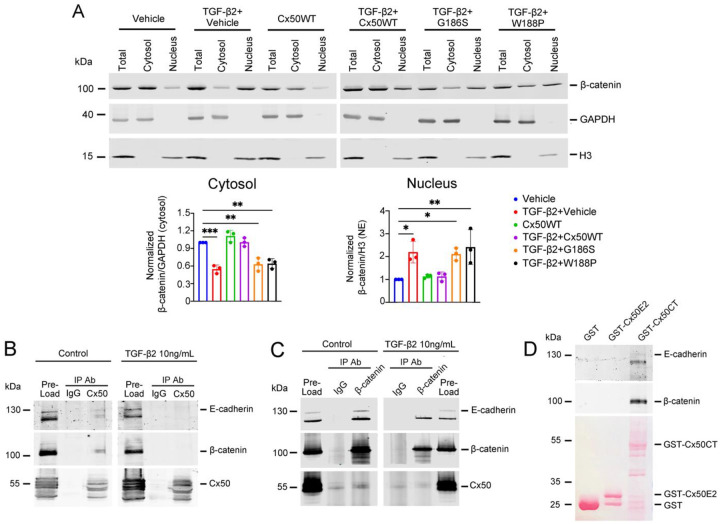
Cx50 prevents nuclear localization of β-catenin through its interaction with β-catenin and E-cadherin via the Cx50CT domain. **(A)** Primary chicken lens cell cultures were infected with recombinant retroviruses expressing WT Cx50 or E2 mutants (G186S and W188P) and treated with 10 ng/mL TGF-β2. Cytosolic and nuclear fractions were separated and immunoblotted for β-catenin, GAPDH, and H3 (upper panels). Band intensities were quantified using NIH ImageJ (lower graphs). Data are presented as mean ± SD (n = 3). *, P < 0.05; **, P < 0.01; ***, P < 0.001 (One-way ANOVA with Tukey’s multiple comparison test). **(B, C)** Primary chicken lens cell cultures were treated with 10ng/mL TGF-β2, and co-immunoprecipitation (CoIP) assays were performed using antibodies for Cx50, β-catenin, and E-cadherin. **(B)** Immunoprecipitation with IgG or anti-Cx50 antibody was followed by immunoblotting for E-cadherin, β-catenin, and Cx50. **(C)** Immunoprecipitation with IgG or anti-β-catenin antibody was followed by immunoblotting for E-cadherin, β-catenin, and Cx50. **(D)** GST-Cx50E2 and GSTCx50CT fusion proteins were used for a protein pull-down assay, and pull-down samples were immunoblotted for E-cadherin, β-catenin and Cx50 (upper panels). GST, GST-Cx50E2 fusion protein, and GST-Cx50CT fusion protein were detected by Ponceau staining (lower panel, pink bands).

To investigate how Cx50 retains β-catenin in the cytosol, primary CLCs were infected with recombinant RCAS(A) retroviruses expressing either WT Cx50 or Cx50 E2 mutants (G186S and W188P), and cytosolic and nuclear fractions were prepared. Western blot analysis showed that β-catenin was predominately present in the cytosolic fraction under basal conditions but became enriched in the nuclear fraction following treatment with 10 ng/mL TGF-β2 (Figure 6A). However, expression of exogenous WT Cx50 reduced β-catenin nuclear localization, while the Cx50 E2 mutants diminished this effect, resulting in increased β-catenin nuclear accumulation. Co-immunoprecipitation using antibodies against either Cx50 (Figure 6B) or β-catenin (Figure 6C) confirmed that Cx50 interacts with both β-catenin and E-cadherin in CLCs. This interaction, however, was disrupted upon treatment with TGF-β2. Additionally, a protein pull-down assay using a GST fusion protein containing the Cx50 CT domain demonstrated the presence of β-catenin and E-cadherin in the pulled-down samples, but not in the GST-only controls (Figure 6D). The results suggest that Cx50 prevents β-catenin nuclear translocation through direct interaction with β-catenin and E-cadherin via its cytosolic CT domain.

### Deletion of Cx50 increases EMT, β-catenin nuclear translocation, and macrophages in an invivo PCO mouse model.

**Figure 7.**
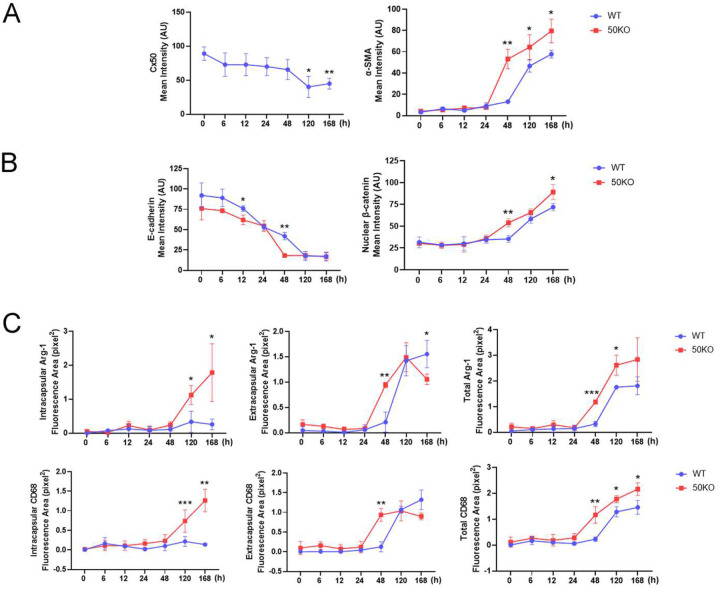
Quantification of EMT marker expression, E-cadherin loss, β-catenin nuclear localization, and macrophage recruitment following Cx50 deletion in an *in vivo* PCO mouse model. Quantification of mean immunofluorescence intensities corresponding to images shown in Supplemental Figure S8. **(A)** Quantification of mean fluorescence intensity of Cx50 and α-SMA at various time points following ECCE surgery in WT and Cx50 KO mice. **(B)** Quantification of mean fluorescence intensity of E-cadherin and nuclear β-catenin. **(C)** Quantification of intracapsular, extracapsular, and total fluorescence area for the macrophage markers Arg-1 and CD68. iNOS expression was undetectable and therefore not quantified. Data are presented as mean ± SD (n = 3). *, P < 0.05; **, P < 0.01; ***, P < 0.001 (unpaired two-tailed t-test).

We mimicked extracapsular cataract extraction (ECCE) in mice, as previously reported [21], using both WT and Cx50 KO mice. Co-immunostaining showed that Cx50 was expressed in LECs from 0 to 48 hours post-surgery in WT mouse, but its expression significantly decreased by days 5 and 7. In contrast, α-SMA expression markedly increased at days 5 and 7, with no detectable α-SMA before day 5 in WT lenses. Compared to WT mice, Cx50 KO mice exhibited significantly higher α-SMA expression as early as 48 hours post-surgery, indicating earlier and more robust EMT induction. α-SMA levels remained significantly higher in Cx50 KO lenses than in WT at all later time points (Figure 7A, **Figure S5, Figure S8**). β-catenin and E-cadherin co-localized at the cell membrane in both WT and Cx50 KO mice. In WT mice, β-catenin and E-cadherin were detectable at 48 hours, although some β-catenin translocated from the membrane to the nucleus at that time point. Notably, β-catenin nuclear localization was significantly greater in Cx50 KO mouse lenses than in WT mouse (Figure 7B, **Figure S6, Figure S8**). No obvious M1 (iNOS-positive) or M2 (Arg-1-positive) macrophages were detected at early time points in either genotype. However, M2 macrophages emerged by 48 hours post-surgery in Cx50 KO mice but were absent in WT mice. Furthermore, Arg-1 expression, a marker of M2 microphages involved in tissue fibrosis, was significantly elevated in Cx50 KO mice compared to WT (Figure 7C, **Figure S7, Figure S8**). These results suggest that Cx50 suppresses EMT progression, β-catenin nuclear localization, and M2 macrophage differentiation, thereby potentially limiting post-surgical fibrosis.

## Discussion

During the EMT process, LECs lose their epithelial characteristics and acquire a fibrotic phenotype. To model this process, we used two PCO *in vitro* and *ex vivo* models using embryonic chick lenses. The *ex vivo* embryonic chick lens wound-repair model has been established for studying LECs migration and PCO progression [22]. We observed that Cx50 expression is inversely correlated with EMT marker expression, suggesting a potential inhibitory role for Cx50 in EMT development. Cx50 is known to be expressed in both lens epithelial cells and fiber cells [7a, 23]. Like other Cx family members, Cx50 contains four conserved transmembrane domains, two extracellular loops, and variable intracellular loop and cytoplasmic CT domains [24]. Our previous research showed that Cx50, unlike Cx43 and Cx46, plays a key role in promoting cell-cell adhesion [11b]. We later identified the E2 domain as the critical mediator of this adhesion-promoting function [11a, 12]. Prior research has shown that enhancing cell-cell adhesion can suppress cell migration and EMT progression [16c, 25]. Based on this, we hypothesized that Cx50 inhibits LEC migration, EMT, and PCO progression by promoting cell adhesion through its E2 domain.

In the *ex vivo* wound-repair model, we observed that by the third day post-surgery, LECs had filled the entire capsule. By the sixth day, the capsule showed signs of wrinkling, and cells not only migrated towards the posterior capsule but also proliferated and extended beyond the capsule’s boundary. Interestingly, α-SMA expression first appeared in the cells that had migrated outside the capsule. As the repair progressed, α-SMA expression increased gradually from the anterior to the posterior capsule, with the highest levels observed in the anterior capsule. These phenotypic changes closely mirror clinical observations [1b]. We further found that EMT markers, including fibronectin and α-SMA, were significantly upregulated by day 6. In contrast, Cx50 and E-cadherin were elevated on day 3, but showed markedly downregulation by day 6. Meanwhile, β-catenin levels gradually increased and were significantly elevated by day 6. During the first three days, the predominant cellular activity involved LEC migration and proliferation, which coincided with increased Cx50 and E-cadherin expression, proteins associated with epithelial integrity and proliferation [26]. By day 6, however, LECs exhibited clear signs of transdifferentiation, characterized by the loss of epithelial markers and a significant reduction in Cx50 and E-cadherin levels. Notably, in these transdifferentiated cells, β-catenin was primarily localized in the nucleus, consistent with its role in EMT. Interestingly, β-catenin expression remained changed in our i*n vitro* cell model, whereas it was significantly increased in the *ex vivo* model. This discrepancy may reflect the active epithelial cell proliferation occurring in the *ex vivo* system, in contrast to the relatively quiescent primary chick lens cultures treated with TGF-β1.

Given that Cx50 expression decreases as LECs undergo EMT, we further investigated whether Cx50 directly regulates LEC migration and the EMT process. In the *in vitro* PCO model using embryonic chick lens cultures treated with TGF-β1, we found that overexpression of Cx50 significantly inhibited cell migration. In contrast, expression of a dominant-negative adhesion-deficient Cx50 E2 mutant reversed the inhibitory effect of Cx50 on cell migration. Similarly, Cx50 overexpression significantly reduced the expression of EMT markers, including fibronectin and α-SMA, while having no effect on E-cadherin levels. However, the Cx50 E2 mutant abolished Cx50’s suppressive effects on fibronectin and α-SMA but did not alter E-cadherin. In the *ex vivo* embryonic chick lens wound-repair model, Cx50 overexpression markedly inhibited LEC migration from the anterior to the posterior, as well as beyond the capsule boundary. By the sixth day post-surgery, compared to the control group, the lenses overexpressing Cx50 maintained a smooth and flat capsule morphology. In contrast, lenses expressing the Cx50 E2 mutants exhibited significantly increased cell migration, accompanied by morphological changes in the capsule, including wrinkling and features of fibrosis. Moreover, Cx50 overexpression significantly reduced the number and extent of α-SMA-positive cells in the lens capsule, while promoting the expression of epithelial cell adhesion markers. These effects were abolished by the Cx50 E2 mutants, indicating that the E2 domain is critical for Cx50’s function. Collectively, these findings suggest that Cx50 inhibits LEC migration and EMT progression through its E2 domain–mediated cell adhesion, in both *in vitro* and *ex vivo* PCO models.

We further investigated whether Cx50 KO affects EMT progression in LECs using both *ex vivo* capsule explants and an *in vivo* PCO mouse model. The *ex vivo* PCO model using cultured mouse lens capsule explants is a widely used approach for studying PCO pathogenesis [27]. We found that Cx50 KO promoted EMT, as evidenced by increased nuclear translocation of β-catenin and loss of E-cadherin expression. Following our *in vitro* and *ex vivo* studies in chick and mouse models, we examined the role of Cx50 in PCO development using an *in vivo* ECLE mouse model, which mimics human cataract surgery by removing fiber cells. In this model, the onset of PCO occurred earlier and was more severe in Cx50 KO mice compared to WT controls. Correspondingly, loss of E-cadherin and nuclear translocation of β-catenin were detected earlier and to a greater extent in Cx50 KO lenses, indicating enhanced EMT progression.

Macrophage infiltration, previously reported to correlate with PCO development [28], was also assessed in our *in vivo* model. During the early stages of PCO, macrophages were largely absent in both WT and Cx50 KO lenses. However, macrophages recruitment occurred earlier and in greater numbers in the Cx50 KO mice, consistent with the increased severity of PCO in these mice. Interestingly, majority of macrophages were of the M2 subtype at both early and late stages, which is indicative of a pro-fibrotic environment. This M2 predominance aligns with the fibrotic nature of PCO and suggests that macrophage polarization contributes to disease progression. Additionally, we observed a spatial pattern in macrophage distribution, with a higher density of cells at the periphery of the lens capsule compared to the central region. These findings suggest that Cx50 plays a suppressive role in microphage infiltration and fibrosis during POC progression, potentially by preserving epithelial integrity and delaying EMT initiation in lens epithelial cells.

We identified a potential molecular mechanism by which Cx50 exerts its inhibitory effects on cell migration and EMT, which appears to be closely linked to its interaction with β-catenin and E-cadherin. Cx50 was found to co-localize with both β-catenin and E-cadherin on the plasma membrane of primary CLCs. Overexpression of Cx50 preserved the membrane localization of β-catenin and E-cadherin and inhibited the nuclear translocation of β-catenin during PCO. However, the dominant-negative adhesion-deficient Cx50 E2 mutants disrupted these effects, leading to reduced E-cadherin expression and increased nuclear translocation of β-catenin. Subcellular fractionation further confirmed that Cx50 retains β-catenin in the cytoplasm, preventing its translocation to nucleus in PCO models. This effect was lost in cells expressing the Cx50 E2 mutant, which allowed β-catenin to accumulate in the nucleus. A likely explanation is that the cell adhesion function of Cx50 stabilizes the E-cadherin and β-catenin complex at the plasma membrane. Supporting this, co-immunoprecipitation assays demonstrated that Cx50 can simultaneously interact with both E-cadherin and β-catenin. Further analysis revealed that the CT domain of Cx50 mediates these interactions. Interestingly, the interaction between Cx50 and β-catenin appeared stronger than that between Cx50 and E-cadherin, suggesting that the Cx50 CT domain may directly bind β-catenin, which in turn associates with E-cadherin to form a tripartite complex. Previous studies have shown a direct interaction between Cx43 and β-catenin [29], and cross-regulation between Cx43 and β-catenin has been implicated in several cellular processes, including proliferation and differentiation [30]. In contrast, our study demonstrates that Cx50’s cell-adhesion function, along with its ability to retain β-catenin at the plasma membrane, plays a critical role in suppressing EMT and PCO development.

**Figure 8.**
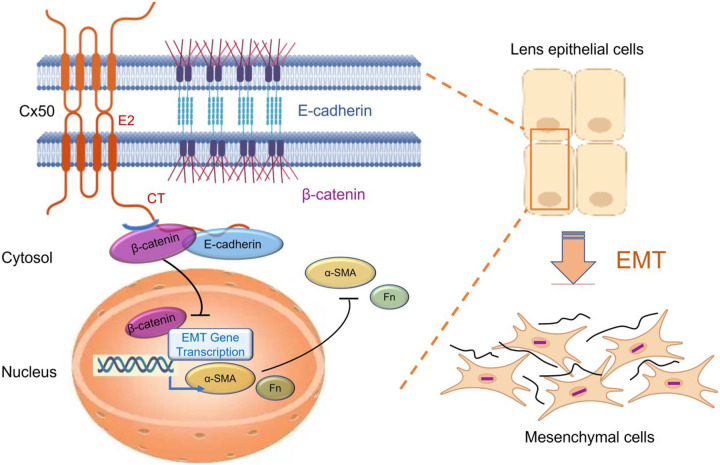
Diagram illustrating the role of Cx50 in suppressing EMT during PCO. Cx50 enhances cell-cell adhesion in LECs through its extracellular E2 domain, thereby inhibiting cell migration. Cx50 interacts with key EMT markers, E-cadherin and β-catenin, helping to preserve E-cadherin expression and retains β-catenin in the cytosol, thus preventing its nuclear translocation. This leads to the inhibition of transcription of genes critical for EMT, including α-SMA and fibronectin (Fn). Furthermore, the downregulation of expression of EMT- and ECM-related genes and protein expression reduces the occurrence of PCO.

In summary, as illustrated in Figure 8, this study using complementary *in vitro*, *ex vivo*, and *in vivo* PCO models demonstrates a unique role of Cx50 and its extracellular E2 domain in inhibiting cell migration and PCO through its cell adhesion function. The CT domain of Cx50 interacts with key cell adhesion proteins, E-cadherin and β-catenin, thereby preserving E-cadherin expression and preventing the nuclear translocation of β-catenin. This retention of β-catenin at the plasma membrane limits the transcription of EMT-related genes such as α-SMA and fibronectin, ultimately inhibiting EMT and the development of PCO.

## Materials and Methods

### Materials

Fertilized White Leghorn chicken eggs were obtained from Texas A&M University, Department of Agriculture & Poultry Science (College Station, TX, USA) and incubated in a humidified chicken egg incubator at 37°C. Rabbit and mouse anti-chick Cx50 antibodies were generated and affinity purified as previously described [31]. Mouse anti-α-SMA antibody (M085129–2) was obtained from Agilent (Santa Clara, CA, USA). Rabbit anti-chicken β-catenin (71-270-0), rabbit anti-H3 antibody (PIPA527029), mouse anti-GAPDH antibody (AM4300), 0.25% Trypsin-EDTA solution, and penicillin/streptomycin were obtained from Invitrogen (Carlsbad, CA, USA). Rabbit anti-FLAG tag antibody (600-401-383) was obtained from Rockland Immunochemicals (Pottstown, PA, USA). Mouse anti-E-cadherin antibody (BDB610181), mouse anti-β-actin antibody (PIMA515739), rabbit anti-iNOS polyclonal antibody (NB300605), IRDye 680RD donkey anti-mouse (NC0250903) and IRDye 800CW donkey anti-rabbit (NC9523609), and Medium 199 (11-150-059) were obtained from Fisher Scientific (Pittsburgh, PA, USA). Mouse anti-fibronectin antibody (610077) was obtained from BD Biosciences (San Jose, CA, USA). Mouse anti-FLAG monoclonal antibody (F1804) was obtained from Sigma (St Louis, MO, USA). Alexa-Fluor-conjugated goat anti-Arg-1 polyclonal (NB10059740) was obtained from Novus Biologicals (Centennial, Colorado, USA.). Rat anti-mouse CD68 antibody (MCA1957) and Nitrocellulose membrane were obtained from Bio-Rad (Hercules, CA, USA). Fluor^®^ 488, 594 or 647-conjugated donkey anti-rabbit antibodies (711-006-152, 711-585-152 or 711-606-152) and donkey anti-mouse antibodies (715-006-150 or 715-586-150) were obtained from Jackson ImmunoResearch (West Grove, PA). Paraformaldehyde (PFA), 16% was obtained from Electron Microscope Science (Fort Washington, PA, USA). Fetal bovine serum (FBS) was obtained from Hyclone Laboratories (Logan, UT, USA). All other chemicals were obtained from either Sigma (St Louis, MO, USA) or Fisher Scientific (Pittsburgh, PA, USA).

### Preparation of high-titer recombinant retroviruses for expression of exogenous proteins

CEF cells were prepared as previously described. Recombinant retroviral DNA constructs and high titer retroviruses containing chicken Cx50 and Cx50 E2 mutants were prepared based on our previous protocol [32]. Primers used for Cx50 and Cx43E2 mutants: Cx50: Sense: 5’−3’CCCACAGAGAAGACCATCTT −3’; Antisense: 5’- CATACAGGAAATACTGGCCAAC −3’. Cx50 E2 mutant G186S: Sense: 5’- TCCCCCTTTACCGCTGTAGCCGGTGGCCC –3’; Antisense: 5’- GGGCCACCGGCTACAGCGGTAAAGGGGGA −3’. Cx50 E2 mutant W188P: Sense: 5’- CCGCTGTGGGCGGCCGCCCTGTCCCAAC −3’; Antisense: 5’-GTTGGGACAGGGCGGCCGCCCACAGCGG −3’.

### Cell culture and immunofluorescence

All animals were housed and studied in accordance with NIH Animal Care and Use Committees (ACUC) guidelines, and the animal protocols were approved by the Institutional Animal Care and Use Committee (IACUC) of the University of Texas Health Science Center at San Antonio (UTHSCSA). Primary chick lens cell cultures were prepared by a modified method described previously [33]. Lenses from embryonic day 11 (E11) chick embryos were isolated, washed with TD buffer (140 mM NaCl, 5 mM KCl, 0.7 mM Na_2_HPO_4_, 5 mM glucose, and 25 mM Tris, pH 7.4), and digested into individual cells using 0.1% trypsin in TD buffer at 37°C. The cells were collected, resuspended in M199 medium supplemented with 10% FBS, and counted. Living cells were then seeded at 2 × 10^5^ cells/cm^2^. On the 2nd day of cell culture, cells were infected with RCAS(A) retrovirus. The cultures were maintained at 37°C with 5% CO2 and the medium was changed every two days. Primary culture cells were treated with recombinant human TGF-β2 (302B2–002).

For immunofluorescence, primary cells were seeded in on glass coverslips until confluent, rinsed with PBS, and fixed in 2% PFA at room temperature (RT) for 30 min. The cells were then blocked with a blocking solution containing 2% goat serum (or serum from another animal source depending on the secondary antibody), 1% BSA, 2% fish skin gelatin, and 0.25% Triton X-100 in PBS. The primary antibody was detected using donkey anti-rabbit, anti-mouse, or anti-goat conjugated with Alexa Fluor 594 or 488 or 647. The samples were then examined under a fluorescence microscope.

### Preparation of crude cell and membrane extracts and western blot

Confluent primary cells were lysed and homogenized with ice-cold lysis buffer (5 mM Tris, 5 mM EDTA, and 5 mM EGTA) containing proteinase inhibitors (5 mM NEM, 2 mM PMSF, 1 mM Na_3_VO_4_, and 0.2 mM leupeptin). The whole-cell lysate was either boiled in 1% SDS or used for preparation of crude membrane extracts. For preparation of membrane extracts, whole cell lysate was homogenized by passing it through a 20G needle and centrifuged at 1000 rpm for 5 min to remove cell debris. The supernatant was then centrifuged at 45,000 rpm for 30 min at 4°C (TLA55 rotor, Beckman Coulter, Brea, CA, USA). The resulting crude membrane pellets were resuspended in lysis buffer containing protease inhibitors and then boiled in 1% SDS. The protein concentration in whole-cell lysates and membrane extracts was quantified using the microBCA assay (Pierce, Rockford, IL, USA) and equal amounts of proteins were loaded onto SDS-PAGE gel. Primary antibodies were detected with goat anti-rabbit IgG conjugated to IRDye^®^ 800CW or goat anti-mouse IgG conjugated to IRDye^®^–680RD (1:15,000 dilution) with a Licor Odyssey Infrared Imager (Lincoln, NE, USA). The intensity of bands on western blots was quantified by NIH ImageJ.

### Extraction of cell cytosol and nuclear proteins from primary lens cells

Protein extraction from primary lens cells was performed using the NE-PER^™^ Nuclear and Cytoplasmic Extraction Reagents (78833, Thermo Fisher) following the manufacturer’s protocol. Briefly, cells were collected using trypsin-EDTA, centrifuged at 500 × g for 5 min, and resuspended in PBS. The cells were then transferred to a 1.5 mL microcentrifuge tube and pelleted by centrifugation. After removing the supernatant, the pellet was resuspended in ice-cold CER I buffer, followed by vigorously vertexing for 15 seconds to fully resuspend the pellet. After centrifugation, the pellet was then resuspended in ice-cold CER II again, vortexed for 5 seconds, and incubated on ice for 1 min. The supernatant was centrifuged at ~16,000 × g for 5 min, and then cytoplasmic extract was immediately transferred to a clean pre-chilled tube. To extract nuclear proteins, the remaining pellet (containing nuclei) was resuspended in ice-cold NER buffer and vortexed for 15 seconds. Vortexing was repeated every 10 min for a total of 40 min. The sample was then centrifuged at ~16,000 × g for 10 min, and the nuclear extract supernatant was immediately transferred to a clean pre-chilled tube.

### Coimmunoprecipitation assay

For immunoprecipitation experiments, primary lens cells were treated with TGF-β2, lysed, and homogenized in ice-cold immunoprecipitation buffer (100 mM NaCl, 15 mM EGTA, 15 mM EDTA, 20 mM Na_2_B_4_O_7_, 10 mM NEM, 2 mM PMFS, 0.02% Na azide, and 1% NP-40, pH 8.5). Cell lysates (120 μg per sample) were precleared with protein A/G plus agarose beads (sc2003; Santa Cruz) and pelleted by centrifugation at 1,000 × g at 4°C for 5 min. The supernatants were then incubated overnight at 4°C with anti-Cx50, anti-β-catenin, or rabbit IgG antibody. The complexes were immunoprecipitated by incubation with 120 μL of protein A/G plus agarose beads for 2 hours at RT. Immunoprecipitates were collected in reducing or nonreducing sample buffer for the detection of β-catenin or Cx50, respectively. The samples were separated on 10% SDS-PAGE gels and immunoblotted with anti-Cx50, anti-E-cadherin, or anti-β-catenin antibodies.

### The chick lens *ex vivo* wound-repair model

The chick embryo lens capsular bag model was established as previously described [22]. To prepare *ex vivo* epithelial injury explants, lenses were removed from the eyes of embryonic day 15 (E15) chick embryos. An incision was made in the anterior lens capsule to remove the lens fiber cell, while the lens epithelium remains tightly adherent to the capsule. Additional cuts were made in the anterior region, creating wound edges that allowed the explants to be flattened and pinned to the culture dish cell-side-up. The *ex vivo* epithelial explants were infected with RCAS(A) retroviruses expressing WT Cx50 or Cx50 E2 mutant retroviruses and cultured in M199 medium supplemented with 10% FBS. Migration of lens epithelial cells from the anterior to the posterior aspects of the capsule was monitored using a Keyence BZ-X710 microscope (Osaka, Japan). Wound area was quantified using NIH ImageJ. At different time points, the chick lens capsular explants were rinsed with PBS, fixed in 2% PFA at RT for 30 min, and blocked with a solution containing 2% goat serum, 1% BSA, 2% fish skin gelatin, and 0.25% Triton X-100 in PBS. Primary antibody detection was performed using donkey anti-rabbit, anti-mouse, or anti-goat secondary antibodies conjugated with Alexa Fluor 594 or 488 or 640, and samples were examined under a fluorescence microscope. The fluorescence intensity was quantified by NIH ImageJ.

### The mouse lens capsule explants

Lenses from WT and Cx50 KO mice, aged between 25 and 30 days, were carefully removed and incubated with the anterior side facing up. Lenses were cultured in serum-free M199 medium and treated with or without TGF-β2 for 24 hours. After treatment, the lens explants were rinsed with PBS, fixed in 2% PFA at RT for 30 min, and blocked with a solution containing 2% goat serum, 1% BSA, 2% fish skin gelatin, and 0.25% Triton X-100 in PBS. Primary antibodies were detected using donkey anti-rabbit, anti-mouse, or anti-goat secondary antibodies conjugated with Alexa Fluor 594, 488 or 640 and examined under a fluorescence microscope.

### The mouse extracapsular lens extraction and preparation of the frozen section

Three-month-old WT and Cx50 KO mice were anesthetized with 100 mg/kg ketamine and 16 mg/kg xylazine. The right eye of each mouse was dilated using several drops of topical tropicamide. An incision was made in the center of the cornea using a Micro Surgical Knife 15° (101413–486, VWR, Radnor, PA, USA), followed by a similar sized incision in the anterior lens capsule. A 26G needle was used to instill saline into the capsular space for hydro-dissection, separating the lens fiber mass from the capsule. The lens nucleus was then removed by applying gentle pressure near the equator of the eye. After expelling the lens mass, the capsule was carefully irrigated to remove any residual lens material, particularly the lens cortex. A viscoelastic agent, hydroxypropyl methylcellulose (75811–344, Spectrum Chemical, New Brunswick, NJ, USA), was then injected into the capsule and anterior chamber to reinflate the eye and maintain structural integrity postoperatively. The corneal incision was closed using 10–0 nylon sutures. Mice were sacrificed at different time points post-surgery. Dissected mouse eyeballs were kept in PBS and a small incision was made using a BD Lo-Dose U-100 Insulin Syringe needle. The entire eyeball was then fixed in 0.75% PFA for 24 hours at RT, followed by sequential dehydration in sucrose gradient solutions: 10% for 1 hour, 20% for 1 hour, and 30% overnight at 4°C. Samples were then embedded in OCT compound (Sakura, Torrance, CA, USA). Sagittal sections (16 μm) were immunostained with primary antibodies overnight at 4°C, followed by incubation with fluorescein-conjugated secondary antibodies for 1 hour at RT and DAPI for 5 min at RT. After rinsing three times with PBS, a drop of mounting medium was added before covering with coverslips. Images were captured using a fluorescence microscope (Keyence BZ-X710), with acquisition settings kept consistent for all samples.

### Statistical analysis

All data were analyzed using GraphPad Prism 7 software (GraphPad Software, La Jolla, CA). Oneway ANOVA followed by Tukey multiple comparison test was used for multiple group comparison. Two-way ANOVA followed by Sidak multiple comparison was used to compare mean differences between two independent variables groups. Data are presented as mean ± SD of at least three measurements. A P-value of <0.05 was considered a statistically significant difference. In all figures, a hashtag (#) indicates significant differences between two independent variables groups. An asterisk (*) indicates the degree of significant differences compared to controls, where *, P< 0.05; **, P < 0.01; ***P<0.001; ****, P < 0.0001.

## Supplementary Material

Supplementary Files

This is a list of supplementary files associated with this preprint. Click to download.

- OriginalDatafiles.pdf
- Supplementaldata.pdf
